# Peripheral Nervous System Interfaces: Invasive or Non-invasive?

**DOI:** 10.3389/fnbot.2022.846866

**Published:** 2022-04-29

**Authors:** Claudio Castellini

**Affiliations:** ^1^Chair of Medical Robotics, Friedrich-Alexander Universität Erlangen-Nürnberg, Erlangen, Germany; ^2^The Adaptive Bio-Interfaces Group, German Aerospace Center (DLR), Institute of Robotics and Mechatronics, Oberpfaffenhofen, Germany

**Keywords:** neurorobotics, machine learning, human-robot interaction, intent detection, somatosensory feedback

## Introduction

Rehabilitation robotics, prosthetics, and assistive robotics are a hard business—especially, *controlling* a rehabilitation robot or a prosthesis is difficult, not only because the user needs to learn to use the device and, possibly, re-learn to use his own body, but also because designing, building, and testing the related control system is difficult. How can a person with no hands, or with severely reduced mobility, let the device know-how and when to grasp, stand, or help perform a reaching movement? Whenever the user's residual ability permits it, an appealing solution, consisting of gathering signals from (intent detection, ID) and stimulating (somatosensory feedback, SF), the peripheral nervous system (PNS), in order to build a bidirectional human-machine, interfaces fostering a deep, synergistic connexion between user and machine (Beckerle et al., 2018).

Now, one of the major hurdles in this field is the generally bad quality of the signals one is interested in gathering and using. Signals coming from the PNS of an impaired user are, *per se*, less stable, reliable, and repeatable than those obtained by able-bodied subjects; moreover, signals gathered in controlled conditions, e.g., while the user sits in a laboratory, can differ dramatically from those the same user produces while trying to perform the same actions in real life (Jiang et al., 2012), hampering the translational potential of research. This problem is being tackled by introducing machine learning able to progressively couple with the user over time (Hahne et al., 2015), allowing for direct, continuous, and sustained interaction with him (Nowak et al., 2018) and by shifting the focus from off-line lab testing to early deployment on end-users in the clinics. There is, however, a second major inherent problem: PNS signals are hard to find. Either we try and build a direct connection to the nerves and/or the muscles (invasive approaches) or we listen to their surface manifestation (non-invasive approaches). Invasiveness means better signals at the price of surgery, to various levels of discomfort for the patient; non-invasiveness is user-friendly and won't violate bodily integrity but yields less clear distorted signals. So, we face a trade-off between the quality of the signals and the quality of user life.

As electronics are more and more integrated with the body, however, more acceptable minimally invasive surgery and permanent implants appear, and their safety and reliability grow. So, eventually, tighter *physical* integration of man and machine could become tolerable and desirable. In a recent perspective paper, (Farina et al., 2021) concur that, at least in prosthetics, where PNS signal processing is the main means to achieve control, osseointegration, targeted muscle reinnervation, and implanted sensors and stimulators are majorly way ahead toward high-performance bionic limbs. One of the messages of the paper is that as technology advances and brings us closer to the biological integration of man and machine, invasive or minimally invasive approaches become more and more appealing, to the point of, eventually, overcoming non-invasive ones and becoming standard.

In this short paper, on the other hand, I argue that PNS non-invasive techniques for both ID and SF will still be indeed preferable in the mid-term. Given the technological advancements we are witnessing in the fields of bodily surface sensing and stimulation, the advantages non-invasive techniques enjoy will still constitute an unsurmountable gap at least in the decade to come. I first talk about non-invasive versus invasive techniques in intent detection (the feed-forward path, section Intent Detection), then in somatosensory feedback (the feedback path, section Somatosensory Feedback), and finally I draw some concluding remarks (section Discussion).

## Intent Detection

Control of motorised prostheses *via* “neural” signals started in the 50s, when two surface electromyography (sEMG) sensors were used to determine the speed of opening/closing of a mechanical one-degree-of-freedom gripper (Fougner et al., 2012). This has remained the clinical standard till the mid-2000s, when multi-fingered prosthetic hands started to appear, calling for a more refined form of control, relying on classification (machine learning) applied to sEMG. Despite the promising perspective of the idea though, still, today there are only two commercial ID systems based upon the classification of sEMG: the *Complete Control* by Coapt Engineering^1^ and the *MyoPlus* by Ottobock^2^, thus, relying on 6-8 single sensors and basic classification. This is not surprising; in general, given the huge variety of daily-living situations, the “one-shot” pattern matching (data collection/model building/control) is unfit for the task (Nowak et al., 2018). Moreover, PNS surface methods, to different degrees, all suffer from sensor displacement caused by donning and doffing, short- and mid-term changes in the morphology of the body, variations in skin impedance, and sensor lift-off (Merletti et al., 2011). The reduced number of sensors that can be embedded in a prosthetic socket is usually insufficient to provide a proper view of muscular activity in the residual limb, and alternative approaches such as pressure sensing and ultrasound scanning are still in the academic prototyping phase (Castellini et al., 2014).

More invasive approaches, on the other hand, promise to provide more focussed, clearer signals, and, as the technology of man-machine integration advances, their appeal increases (Farina et al., 2021). PNS invasive techniques entail different degrees of invasiveness, from the injection of miniaturised EMG sensors into the muscles (Weir et al., 2009) to osseointegration (Ortiz-Catalan et al., 2014) and targeted muscle reinnervation (Aszmann et al., 2015); depending on the severity of the impairment then, patients might agree to stand surgery.

Nevertheless, besides the absence of surgery, surface ID approaches rely on technology requiring (almost) no direct interfacing to biological tissue. Improving surface sensing means achieving higher resolution, both in time and in space, and pushing on miniaturisation; and to achieve this, no complex biological interfaces are needed. To me, this means that, for a long time to come, the non-invasive approaches will still maintain a technological advantage over invasive ones. High-density *wearable* sEMG arrays comprising ~120 sensors sampled at 2 kHz have recently appeared (e.g., the *MuoviPro* by OTBioelettronica^3^), as well as ultraminiaturised ultrasound scanning devices, whose transducer array fits in the size of a coin (Fournelle et al., 2021). Both systems deliver an unprecedented precision, both in time and space, in the detection of residual muscle activity.

To sum up, high-density surface approaches, coupled with “life-long” interactive machine learning, are not limited to the one-shot schema (Castellini et al., 2015; Beckerle et al., 2018) and shall overcome the problems that are typically associated to non-invasiveness (low density and unreliability in time) *at no additional burden for the user*. For this reason, I argue, they will still represent the methods of choice in the short- and mid-term.

## Somatosensory Feedback

Similar remarks hold, in my opinion, as far as SF is concerned. By SF in this specific framework mechanical or electrical signals are provided to the user's somatosensory system, which relates to the status of the rehabilitation device and/or the environment (Beckerle et al., 2018). SF enables *closed-loop human control* of rehab- and assistive devices: through repeated, stable and identifiable patterns of SF stimulation, corresponding to actions of the device, the user increases the sense of agency (being in control) and ownership (being a part of the self) over it, leading to embodiment and—at least, this is the current opinion in the community—reciprocal adaptation and optimal control (Hahne et al., 2015). The goal is to provide a rich set of feelings to the user, both in terms of the type of feeling (touch, pressure, temperature, texture, etc.) and of its intensity, responsiveness, and appropriateness. At the same time, the stimulator and control system must be as small, lightweight, and low-power as possible (Došen et al., 2017).

Given these goals and despite the spectacular advancements appearing in the scientific literature in the past years, invasive SF is still far from being clinically applicable. Although experiments are reported, in which direct electrical stimulation of peripheral nerves has enabled users to discriminate several tens of spatially stable sensations, such implants are mostly temporary (remarkable exceptions to this are reported, e.g., in Petrini et al., 2019; Ortiz-Catalan et al., 2020), and involve complex surgical procedures; also, nociception seems to be a non-negligible associated issue (Davis et al., 2016). Non-invasive SF, on the other hand, has recently evolved from bulky sets of a few vibrotactile/mechano-tactile actuators to hundreds of high-density electrical stimulators. Electro-tactile stimulation (ECS), for example, works by injecting small electrical currents across the skin, thereby not affecting muscle contractions, and maintaining compatibility with sensing techniques for intent detection (e.g., sEMG, see Došen et al., 2017). High-density integrated ECS delivers highly differentiated stimuli, both qualitatively and quantitatively, at essentially no additional psychological burden for the user (Dideriksen et al., 2022; Isaković et al., 2022). In my opinion, any advantage delivered by direct connection to the afferent nerves, e.g., cuff or intra-fascicular electrodes [and there is ample evidence for their effectiveness, see for instance (D'Anna et al., 2019; Petrini et al., 2019)], needs to face an inevitable acceptance gap; moreover, here, too, as it was the case for ID, the technologies involved in ECS vs. invasive techniques belong to two different levels of maturity.

## Discussion

This work has concentrated on prosthetics; nevertheless, in my opinion, similar remarks apply to rehabilitation robotics—to a lesser extent, indeed, but in an important way. The usage of ID and SF in the management of rehabilitation exoskeletons, exo-suits, and virtual reality environments is still in its infancy, since robotic therapies for, e.g., stroke survivors and patients with SCI are still largely based upon repetitive motions, not initiated by the patients (if not *via* verbal interaction with a therapist). Here, PNS interfacing is a second-class denizen, although progress in this direction is being made (Lobo-Prat et al., 2014; Sullivan et al., 2017), since, as opposed to the case of prosthetics, patients in rehabilitation are supposed to engage in their therapy only for a limited amount of time and already suffer from neural conditions. For these reasons, it seems to me that ID/SF techniques in rehabilitation should be kept even more non-invasive.

Non-invasive techniques for sensing and stimulation in prosthetics have clear advantages over invasive ones; both in terms of their immediate applicability, but also in perspective, since they do not need direct interfacing with the nervous system. This is just my informed opinion, and this paper is not meant as a survey, and, for sure, does not exhaustively cover the field; so, my statement may be challenged or even overturned in a few years. Still, the technological advancement of non-invasive techniques is faster, and I believe that their advantage over invasive techniques will remain significant for at least one decade; they will still be preferable to invasive ones. Of course, as the technology of invasive techniques progresses, too, there could be a point in the future at which they will be as convenient as non-invasive ones. Mixing invasive and non-invasive techniques could, at that point, be an interesting option to take out the best of both worlds, for instance, coupling ultrasound-based ID and SF are given by implanted stimulators (a thorough review, providing a unified view of such techniques, is found in Shokur et al., 2021). A life-long adaptive form of machine learning will be needed, just as it is today, to reach optimal control of any reha- or assistive device. If it is true that more good data is better, it is also true that data from impaired users change in time, whatever the source of data is, and in that case, a strategy to determine which data to use or to emphasise will be needed. True adaptation to the user should provide this possibility.

## Author Contributions

The author confirms being the sole contributor of this work and has approved it for publication.

## Funding

This work was partially supported by the DFG (German Research Agency) project *Deep-Hand*, CA1389/1-2.

## Conflict of Interest

The author declares that the research was conducted in the absence of any commercial or financial relationships that could be construed as a potential conflict of interest.

## Publisher's Note

All claims expressed in this article are solely those of the authors and do not necessarily represent those of their affiliated organizations, or those of the publisher, the editors and the reviewers. Any product that may be evaluated in this article, or claim that may be made by its manufacturer, is not guaranteed or endorsed by the publisher.

## Footnotes

^1^ https://coaptengineering.com

^2^ https://www.ottobockus.com/prosthetics/upper-limb-prosthetics/solution-overview/myo-plus/myo-plus.html

^3^ https://www.otbioelettronica.it/component/sppagebuilder/?view=page&id=150
